# Facile synthesis of concentrated gold nanoparticles with low size-distribution in water: temperature and pH controls

**DOI:** 10.1186/1556-276X-6-440

**Published:** 2011-07-06

**Authors:** Chunfang Li, Dongxiang Li, Gangqiang Wan, Jie Xu, Wanguo Hou

**Affiliations:** 1 State Key Laboratory Base of Eco-Chemical Engineering, Lab of Colloids and Interfaces, College of Chemistry and Molecular Engineering, Qingdao University of Science and Technology, Qingdao 266042, China

**Keywords:** gold nanoparticles, concentrated, sodium citrate

## Abstract

The citrate reduction method for the synthesis of gold nanoparticles (GNPs) has known advantages but usually provides the products with low nanoparticle concentration and limits its application. Herein, we report a facile method to synthesize GNPs from concentrated chloroauric acid (2.5 mM) via adding sodium hydroxide and controlling the temperature. It was found that adding a proper amount of sodium hydroxide can produce uniform concentrated GNPs with low size distribution; otherwise, the largely distributed nanoparticles or instable colloids were obtained. The low reaction temperature is helpful to control the nanoparticle formation rate, and uniform GNPs can be obtained in presence of optimized NaOH concentrations. The pH values of the obtained uniform GNPs were found to be very near to neutral, and the pH influence on the particle size distribution may reveal the different formation mechanism of GNPs at high or low pH condition. Moreover, this modified synthesis method can save more than 90% energy in the heating step. Such environmental-friendly synthesis method for gold nanoparticles may have a great potential in large-scale manufacturing for commercial and industrial demand.

## Introduction

Gold nanoparticles (GNPs), also named as gold colloids, have attracted increasing attention due to their unique properties in multidisciplinary research fields [1,2]. Although GNPs are defined by tiny size, significant quantities of GNPs are likely required in many commercial and industrial applications. Remarkably, novel emerging applications bring a huge growth of the global demand of GNPs. For instance, (1) biomolecule- and/or biopolymer-conjugated GNPs are largely used as biomarkers and biodelivery vehicles in the medicine/pharmacy, and in cosmetic products, GNPs are employed as anti-aging components for skin protection [3-5]; (2) GNPs are used to treat wool or cotton fibers for a permanent coloration [6] of value textiles; (3) various polymer/gold nanocomposites display a high potential for novel coatings and paintings [7-11]; (4) GNPs are used to enhance the performance of non-volatile memory devices [12] and low temperature printing metal inks in electronics [13]; and (5) GNPs as catalysts are developed in novel usages [14-18]. Therefore, more attention should be paid on effective synthesis methods to match the enlarging demand of GNPs.

In the past decades, though many synthetic strategies have been developed to prepare GNPs in organic or aqueous solvents [19-24], the citrate reduction method has remained the best candidate to fit the enlarging demand of GNPs due to its advantages such as inexpensive reductant, non-toxic water solvent, and low pollution in the reaction [25-28]. The simple operation of pouring rapidly a certain amount of sodium citrate solution into a boiling solution of 0.25 mM chloroauric acid produces narrowly distributed GNPs which are biocompatible and easily handled in applications [29-31]. So, this method is extensively used in GNP-based bioassays and biomedicine systems [5,32-34] and even in structured/assembled nanomaterials [35-41]. In the pioneering work on the citrate reduction method, Turkevich in 1951 reported the basic experimental approach and the effect of temperature and reagent concentration upon the nanoparticle size and size distribution [25], and in 1973, Frens published the control of size variation of GNPs by changing the concentration of sodium citrate [26]. Then, in 1994, Zukoski published a sol formation mechanism and a particle growth model [42]. Recently, the decisive role of sodium citrate on the pH value of the reaction mixture and the nanoparticle size was demonstrated based on experimental and theoretical modeling results [27,43,44]. On the other hand, in the majority of the published citrate reduction works, GNPs were synthesized from a dilute solution of 0.25 mM chloroauric acid, such a concentration yields aqueous GNPs with low weight content (0.005%) as a disadvantage. The low nanoparticle content asks for abundant water to be used in the preparation and consumes a lot of energy in the heating step. Sometimes, such dilute gold colloids cannot fulfill the requirement of high concentration. Thus, the classical citrate method will be limited in large-scale manufacturing. Considering the abovementioned advantages and disadvantages, we expected that the citrate reduction method should have been developed to produce concentrated aqueous GNPs already from several years ago. However, simply increasing the reactant concentration will change the systemic pH and salt concentration with drastic influence on the nanoparticle size polydispersity and the colloidal stability.

Herein, to meet the need of high concentrations, we modified the classical citrate reduction method and synthesized uniform GNPs from tenfold concentrated precursor (2.5 mM HAuCl_4_) via adding sodium hydroxide and controlling the temperature. We demonstrated that adding a proper amount of sodium hydroxide to the reaction mixture could produce uniform GNPs with a narrow size distribution after the reduction by sodium citrate at boiling sate. The low reaction temperature was helpful to control the nanoparticle formation rate, and uniform GNPs could be obtained at different temperature by adding a proper amount of alkali. The pH change resulting from the addition of alkali showed a critical role in the influence on the particle size distribution, which might be related to the different formation mechanism of GNPs under different pH conditions.

## Experimental methods

### Materials

Hydrochloroauric acid trihydrate (HAuCl_4 _3H_2_O, 99.9%) was purchased from Sigma-Aldrich Shanghai Trading Co Ltd, Shanghai, China, while sodium citrate (Na_3_C_6_H_5_O_7 _2H_2_O, > 99%) and sodium hydroxide (NaOH, > 98%) were obtained from Shanghai Chemical Co., Shanghai, China. Deionized water (resistance > 18.2 MΩ) was prepared by an ultrapure water system in our laboratory. All chemicals were used as received without any purification.

### Synthesis of concentrated nanoparticle dispersions via simply increasing reactant concentration

GNPs were first synthesized from HAuCl_4 _solution with gradually increased concentration of the reactant. In detail, 50 ml deionized water in a round-bottom flask was added to 5, 10, 20, 30, 40, and 50 mg chloroauric acid, respectively. After heating to boiling state, 0.3, 0.6, 1.2, 1.8, 2.4, and 3.0 ml sodium citrate solution (50 mg/ml) were rapidly introduced into the flask with drastic stirring, respectively. The mixtures were continuously heated for a certain period till a ruby-red color appeared.

### Synthesis of concentrated GNPs under alkali control and different temperature

The concentrations of chloroauric acid and sodium citrate in the final mixture were respectively fixed to 2.5 and 5.0 mM, while that of NaOH was changed. The reaction temperature was selected to be boiling state, 85°C and 70°C. For example, 2.0 mL chloroauric acid (25 mM) was mixed with 5.3 to 10.2 mL of 20 mM NaOH solution, followed by adding the calculated volume of water to a total volume of 20 mL. The flask was put into an oil bath at 110°C for 30 min to balance the reaction mixture to 85°C. Then, 0.6 mL sodium citrate solution (50 mg/ml) was rapidly introduced into the flask under vigorous stirring. After different reaction time, samples were taken out for characterization. The reaction at the boiling state and 70°C was similarly performed, respectively.

### Detecting the nanoparticle formation process

In the synthesis process of GNPs, a portion of the reaction mixture (0.5 to 1 mL) was taken out from the flask at different reaction time and immediately poured into 9 mL ice-cooled water at 0°C. Such an operation can basically cease the formation process of GNPs due to the low temperature surrounding and the dilution effect, so it was called here as a "sample-frozen" operation. Then, the transmission electron microscopy (TEM) samples were prepared at the earliest time and the ultraviolet-visible (UV-vis) spectra were recorded.

### Characterization and instrumentation

UV-vis spectra were recorded on a U-3010 UV-visible spectrophotometer (Hitachi High-Technologies Co., Tokyo, Japan) to collect the surface plasmon resonance (SPR) information of GNPs, in which the highly concentrated samples were diluted pro rata by deionized water to adapt the measurement limitation. TEM samples were prepared by dropping the diluted gold colloids on carbon-coated copper grids, followed by natural drying; then, the samples were observed on a JEM-2010 microscope (JOEL Ltd, Tokyo, Japan).

## Results and discussion

### Size distribution enlarging of GNPs at high reactant concentration

**Figure 1:**
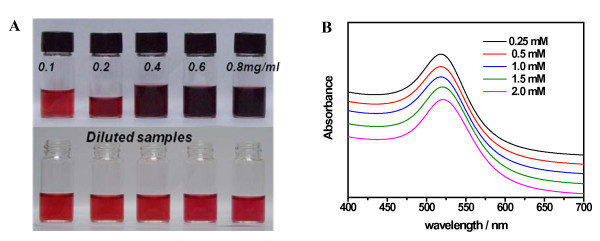
**Optical photos of the obtained gold colloids, the diluted samples and their corresponding UV-vis spectra**. (**A**) Photos of gold colloids prepared from a solution of 0.25 mM to 2.0 mM HAuCl_4 _3H_2_O (corresponding to 0.1 mg/ml to 0.8 mg/ml, respectively) and their diluted samples at a content of 0.25 mM Au. (**B**) The corresponding UV-vis spectra of the diluted samples. (Baseline was adjusted artificially).

**Figure 2:**
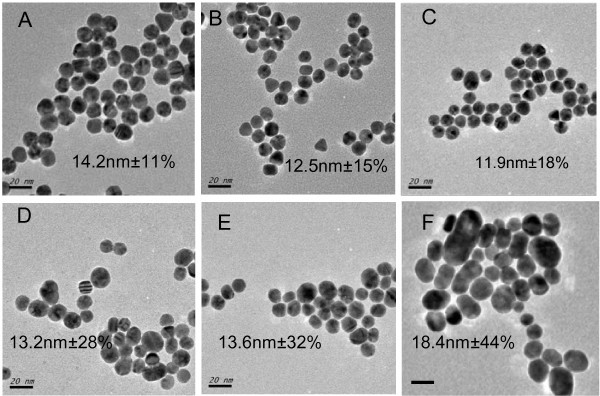
**TEM images of GNPs with indicated size and polydispersity**. They are prepared by conventional citrate method from 0.25 mM (**A**), 0.50 mM (**B**), 1.0 mM (**C**), 1.5 mM (**D**), 2.0 mM (**E**), and 2.5 mM (**F**) chloroauric acid, respectively. Scale bar: 20 nm.

In Turkevich's work, the influence of reactant concentration of HAuCl_4 _from 0.25 mM to decreased values was studied [25]. Herein, our first effort was taken to prepare GNPs through gradual increase of reactant concentration by the classical citrate method. Aqueous chloroauric acid solution from 0.25 to 2.5 mM was heated to boiling and the four times molar amount of sodium citrate was added, followed by continuously heating for a certain period to get the ruby-red colloids. It was found that the reaction rate was greatly enhanced at high reactant concentration. The optical photos of the obtained samples and diluted samples, as well as the corresponding UV-vis spectra, are shown in Figure 1. The color and the surface plasmon resonance (SPR) peaks of these colloids do not show obvious differences, and no obvious difference is found in the full width at half maximum of these peak profiles. However, TEM images of these GNPs (Figure 2) show that the size polydispersity remarkably varies with the reactant concentration increase although the particle average sizes are all located in a range of 10 to 20 nm. The large size distribution of GNPs at high reactant concentration will limit further applications such as size-related bioassays and well-defined nanoassembly. Moreover, the as-obtained gold colloids from 2.5 mM HAuCl_4 _are not stable and become black precipitates after hours; this is partially ascribed to the colloidal instability at high ionic strength.

### Controlling the size distribution by adding sodium hydroxide

**Figure 3:**
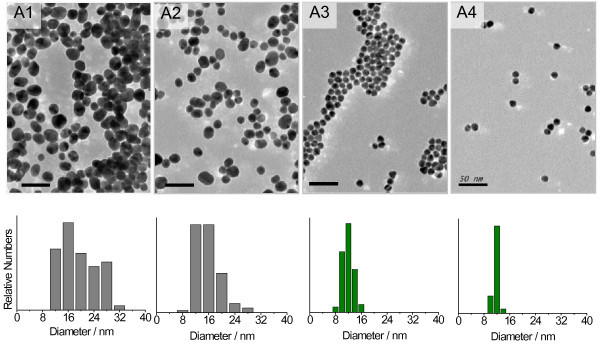
**TEM images and size distribution diagrams of GNPs**. They were synthesized at boiling state under addition of different NaOH content of (A1) 3.1 mM, (A2) 4.4 mM, (A3) 5.3 mM, and (A4) 6.6 mM, respectively. Scale bar: 50 nm.

In recent published work, pH control was reported to produce monodisperse GNPs with low polydispersity [27,43,44]. In our experiments, we found that the increase of the reactant concentration slightly decreased the pH of the final mixture. Thus, we were inspired to add sodium hydroxide (NaOH) into the reaction mixture as a trial to lower particle polydispersity. Then, GNPs were prepared at boiling state with fixed 2.5 mM chloroauric acid and 5 mM sodium citrate (calculated based on the volume of the final mixture). This reduction of the molar ratio of citrate to chloroaurate was applied to decrease the ionic strength in the final colloids. It was found that the reaction rate was reduced as the alkali was added into the reaction system, but precipitates appeared under a high NaOH concentration of 7.8 mM. The color of the obtained colloids was not obviously different from each other (Figure S1 in Additional file 1). Figure 3 shows the TEM images of GNPs synthesized under different NaOH amount from 3.1 to 6.6 mM, and their size distribution was measured from more TEM images as shown below each image. Obviously, the particle size polydispersity was largely decreased with the increase of added NaOH amount. We find that the obtained particles at 5.3 and 6.6 mM NaOH have a narrow size distribution, and the best alkali dosage is 6.6 mM. However, the reaction rate was still found to be too fast to be controlled well, although the alkali's addition could lower it in a certain extent. The time that the color changed to red after adding sodium citrate was still only 1 min in presence of 6.6 mM NaOH, and the reaction flask had to be removed from the oil bath at once, otherwise aggregated particles were obtained (Figure S1 in Additional file 1) possibly due to the kinetic instability [45]. Moreover, at different reaction time, portions of the reaction mixture were taken out and were recorded by UV-vis spectrophotometer. The SPR peaks of these samples (Figure S2 in Additional file 1) show that under the presence of 5.3 and 6.6 mM NaOH, the gold colloids after 1- to 2-min reaction have an SPR peak around 518 nm which corresponds to the uniform colloids. However, at longer reaction time, the SPR peaks are strongly red shifted, indicating an aggregation process in accordance with the TEM results. Therefore, the synthesis time under the boiling state should be no longer than 2 min.

### Decreasing reaction rate by lowering temperature

**Figure 4:**
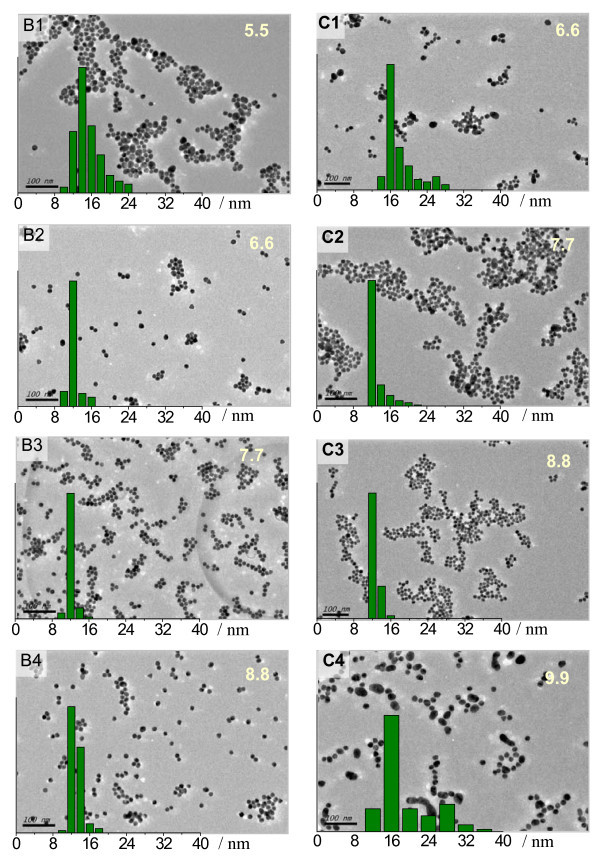
**TEM images and size distribution diagrams of GNPs**. They were synthesized under labeled NaOH concentration (millimolars) at 85°C (B1-B4) and 70°C (C1-C4), respectively. Scale bar: 100 nm.

Basically, the chemical reaction rate drastically depends on temperature, so the high rate of nanoparticle formation can be decreased at a low temperature. In this work, the nanoparticle synthesis was therefore performed at 85°C and 70°C with a defined range of NaOH amount. It was found that the formation rate of GNPs slowed as expected at lower temperatures. The color of the colloids obtained at 85°C (Figure S1 in Additional file 1) did not differ from that of those produced under boiling state. TEM images of the synthesized GNPs at 85°C in the presence of different alkali amount were shown in Figure 4 (B1 to B4), including the particle size polydispersity. We could find that GNPs synthesized in presence of 5.5 mM NaOH have an average size of 15 nm with large size distribution, while at a high NaOH concentration, from 6.6 to 8.8 mM, the particle size was slightly decreased to 12 to 13 nm with a narrow distribution. The best GNPs were produced in presence of 7.7 mM NaOH. The higher NaOH dosage of 9.9 mM could only produce purple-color colloid which was not stable and precipitated after hours. The SPR peaks of the gold colloids taken-out from the reaction mixture at different time were also studied by UV-vis spectroscopy (Figure S2 in Additional file 1). We found that the colloidal samples prepared at 6.6 to 8.8 mM NaOH show SPR peaks around 519 nm, and the reaction time should be controlled at 10 to 15 min, although longer reaction time did not cause aggregation.

**Figure 5:**
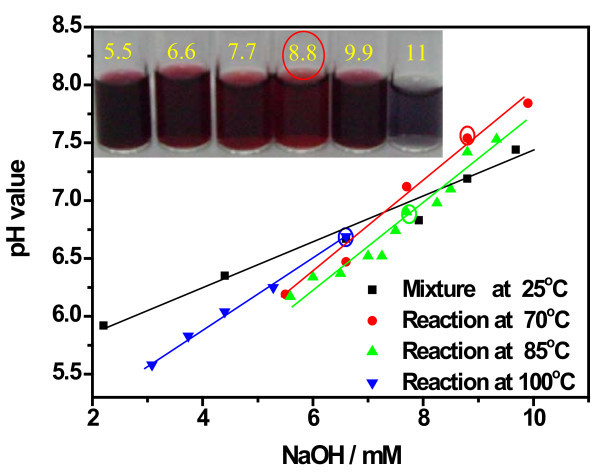
**pH values of Au colloid dispersions obtained at different temperature versus NaOH concentration**. The pH values before reaction were also involved and the inset photo shows Au colloids prepared at 70°C under the labeled alkaline concentration (millimolars).

Similarly, as shown in Figure 4 (C1 to C4), the TEM results of GNPs synthesized at 70°C show the same tendency in particle size and size distribution in presence of different NaOH amount. The dosage of NaOH influences the particle size distribution, and the optimal alkali concentration should be 8.8 mM for the most uniform nanoparticles. The reaction under 9.9 mM NaOH needs a long time heating after citrate addition and produces broadly size distributed GNPs (Figure 4 C4). Optical photos of these gold colloids are shown in the inset of Figure 5. The color of samples prepared under 7.7 and 8.8 mM NaOH is similar, which is slightly different from that of samples prepared at 6.6 and 9.9 mM NaOH. The sample prepared at 5.5 mM NaOH was dark red while that prepared under 11 mM NaOH was cyan due to the aggregation and precipitation of nanoparticles. The SPR peaks (Figure S2 in Additional file 1) of the gold colloids obtained after different reaction times showed that the gold colloids synthesized at optimal conditions (NaOH 7.7 to 8.8 mM) had SPR peaks around 520 nm and the reaction time should be 20 to 25 min.

It can be concluded that uniform GNPs can be synthesized from concentrated gold precursor solution of 2.5 mM based on the citrate reduction by pH and temperature control. The recommended experimental parameters are listed in Table 1. This modified citrate method will largely save energy in the heating stage because of two main reasons. The first is, because the concentration of gold precursor is tenfold compared to the majority of common uses, the usage of only 10% water solvent will save 90% heating energy. Secondly, if the reaction is performed at 70°C or 85°C and room temperature is 25°C, the low temperature reaction will further save 40% or 20% energy, and totally save 94% or 92% heating energy, compared with the dilute concentration and boiling state reaction. Furthermore, it should be noted that the obtained concentrated gold colloids had a good stability, no change was found in the colloid color and the UV-vis absorbance after more than 1-year storage at the room temperature.

**Table 1:** Optimal experimental parameters for GNP synthesis at different temperature

Reaction temperature	NaOH (mM)	Reaction time (min)	Final pH
Boiling state	5.3-6.6	1-2	6.3-6.7
85°C	6.6-8.8	10-15	6.4-7.4
70°C	7.7-8.8	20-25	7.1-7.5

### pH analysis of the reaction mixture at different conditions

Figure 5 displays the pH values of the reaction mixture mixed at room temperature and those as-obtained gold colloids prepared at various conditions. The pH value shows a linear change with respect to the addition of NaOH both before and after the reaction, which is due to the buffer behavior of the sodium citrate and the low alkali dosage. When the reaction was performed at boiling state, the optimal NaOH dosage (6.6 mM) corresponds to pH 6.7. At 85°C, the pH of the best colloids prepared in presence of 7.7 mM NaOH is 6.8, while at 70°C the final pH for the best colloids is 7.5. The pH values of the acceptable GNPs with a narrow size distribution are listed in Table 1. It is found that the pH values for uniform gold colloids are slightly different at different reaction temperatures and a higher pH value is indicated at lower temperature. These pH values are very close to the neutral condition (between 6.5 and 7.5), which is in accordance with the literature [27].

### Analysis of the pH influence on the nanoparticle size distribution

**Figure 6:**
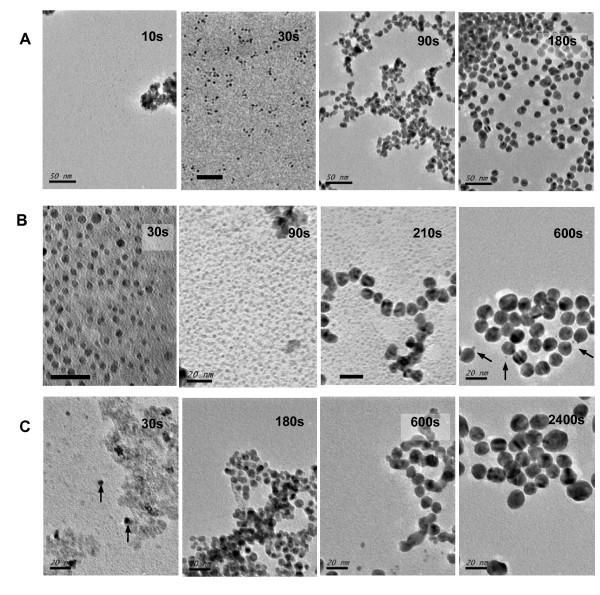
**TEM images of temporal evolution of GNPs after the labeled reaction time**. These samples were obtained from the reaction process at 85°C in the presence NaOH with a concentration of (**A**) 6.0 mM, (**B**) 7.7 mM, and (**C**) 9.5 mM, respectively. Scale bar: 50 nm in (A) and 20 nm in (B, C).

From the above results, the alkali concentration and the pH value should play a critical role in controlling the size distribution of finally synthesized GNPs. To discover the pH effect on nanoparticle formation, we use a so-called frozen method to cease the nanoparticle growth at different reaction time at 85°C as described in the experimental section and investigate the TEM morphology changes and UV-vis spectra. Three NaOH dosages of 6.0 mM (corresponding to a low pH), 7.8 mM (a medium pH, near the optimal condition), and 9.0 mM (a high pH) were used to prepare reaction-time-dependent samples under different pH conditions. UV-vis spectra and photos (Figure S3 in Additional file 1) of the time-dependent samples can only show the macroscopic changes with time, from which only the difference of the reaction rate can be shown under different pH conditions. The microscopic changes in the process of nanoparticle formation are shown by the TEM images in Figure 6. With the addition of 6.0 mM NaOH, many small particles with about 2 nm in diameter were found after 10-s reaction, and then, the particles grew to 4-nm size at 30 s and about 8-nm particles appeared at 90 s. After 180 s, the formed GNPs did not obviously change their shapes (Figure 6A). In case of 7.8 mM NaOH, similarly, many 3-nm small nanoparticles were found after 30 s (Figure 6B). Then, these small particles grew into large ones of about 10 nm at 210 s, and the final particle size was about 14 nm after 10-min reaction. It should be noticed that these 3-nm small particles continuously exist in the whole particle formation process and even in the final samples (arrow marked). This phenomenon was not found in the low pH case, and it is indicated that the nanoparticle growth step is different at low and medium pH. Thus, the difference in the nanoparticle growth step at low and medium pH might result in the difference of the size polydispersity of the final GNPs. Differently, at high pH (9.5 mM NaOH), both the small particles of about 2 nm and the large particles of about 8 nm (arrow marked) were found after only 30-s reaction (Figure 6C). This is obviously different from the low pH conditions (6.0 and 7.8 mM NaOH) and might imply a different nucleation or coagulation step in the nanoparticle formation at high pH which causes the enlargement of the size distribution. Anyway, the nanoparticle formation process at low or high pH is different from that at mediate pH either in the final nanoparticle growth step or in the beginning nucleation/coagulation step. Therefore, the pH influence on the size distribution of GNPs factually reveals the different formation mechanism of GNPs at different pH conditions as mentioned in the literatures [44,46-49].

## Conclusions

In this work, uniform GNPs with low size polydispersity can be synthesized from the chloroauric acid precursor at high concentration (2.5 mM) by the citrate reduction method via combined temperature and pH controls. The addition of a proper amount of sodium hydroxide can produce uniform GNPs with a narrow size distribution. The low reaction temperature is helpful to control the nanoparticle formation rate, and uniform GNPs can be obtained at different temperatures in presence of an optimized NaOH dosage. The pH analysis demonstrates that uniform GNPs can be obtained at around neutral conditions. The modified citrate reduction method can produce concentrated gold colloid dispersions and save more than 90% energy in the heating step. Such environmental-friendly synthesis method for gold nanoparticles may have a great potential in large-scale manufacturing to match the increasing commercial and industrial demands.

## Competing interests

The authors declare that they have no competing interests.

## Authors' contributions

CL and GW took the tasks of experimental, basic data collection, and the draft writing; DL gave his contributions on the experimental guidance and TEM observation, as well as the main paper organization; JX took some spectrometric works; and WH took the contributions on the research guidance, discussion, and paper modification.

## Authors' information

DL is a Ph.D. major in Physical Chemistry, Shandong University, China. He has focused his research interest on the gold nanomaterials especially on the polymer modified gold nanoparticles for more than 6 years from his postdoc careers in Institute of Chemistry, Chinese Academy of Sciences, China and in the Max-Planck Institute of Colloids and Interfaces, Germany. His published papers involved the core/shell nanostructures of the thermosensitive/pH-responsive polymer and amphiphilic polymer grafted gold nanoparticles toward multifunctional nanocarriers and nanosupports.

## Supplementary Material

Additional file 1 **Sample photos, supplementary TEM images, SPR peak changes and UV-vis spectra**. Sample photos of concentrated GNPs prepared at different conditions, supplementary TEM images of a selected sample of aggregated Au colloids, SPR peak changes of gold colloids prepared after different reaction time, and the temporal changes of UV-vis spectra and photos in the formation process of GNPs. Click here for file
