# Supplementary material for: Facile synthesis of concentrated gold nanoparticles with low size-distribution in water: temperature and pH controls
Source: Nanoscale Res Lett. 2011 Jul 6;6(1):440. doi: 10.1186/1556-276X-6-440 (PMC3211859; doi:10.1186/1556-276X-6-440)
Supplement: Additional file 1 — Sample photos, supplementary TEM images, SPR peak changes and UV-vis spectra. Sample photos of concentrated GNPs prepared at different conditions, supplementary TEM images of a selected sample of aggregated Au colloids, SPR peak changes of gold colloids prepared after different reaction time, and the temporal changes of UV-vis spectra and photos in the formation process of GNPs. [file 1556-276X-6-440-S1.DOC]

**Sample photos, supplementary TEM images, SPR peak changes and UV-vis spectra.**

**Additional Supporting Materials**

*for*

**Facile synthesis of concentrated gold nanoparticles with low size-distribution in water: temperature and pH controls**

Chunfang Li^1^, Dongxiang Li*^1^, Gangqiang Wan^1^, Jie Xu^1^ and Wanguo Hou*^1^

^1^State Key Laboratory Base of Eco-Chemical Engineering, Lab of Colloids and Interfaces, College of Chemistry and Molecular Engineering, Qingdao University of Science and Technology, Qingdao 266042, China.

*Corresponding authors: [lidx@iccas.ac.cn](mailto:lidx@iccas.ac.cn), [wghou@sdu.edu.cn](mailto:wghou@sdu.edu.cn)

**Figure S1.** (A) Photo of gold colloids prepared in boiling state in presence of marked NaOH concentration (mM). (B) Big TEM image corresponding to Figure 3 A4 (left) and TEM image of the aggregated Au colloids after 16 min boiling in presence of 6.6 mM NaOH (right). (C) Photo of gold colloids prepared at 85 ^o^C in presence of marked NaOH concentration (mM).


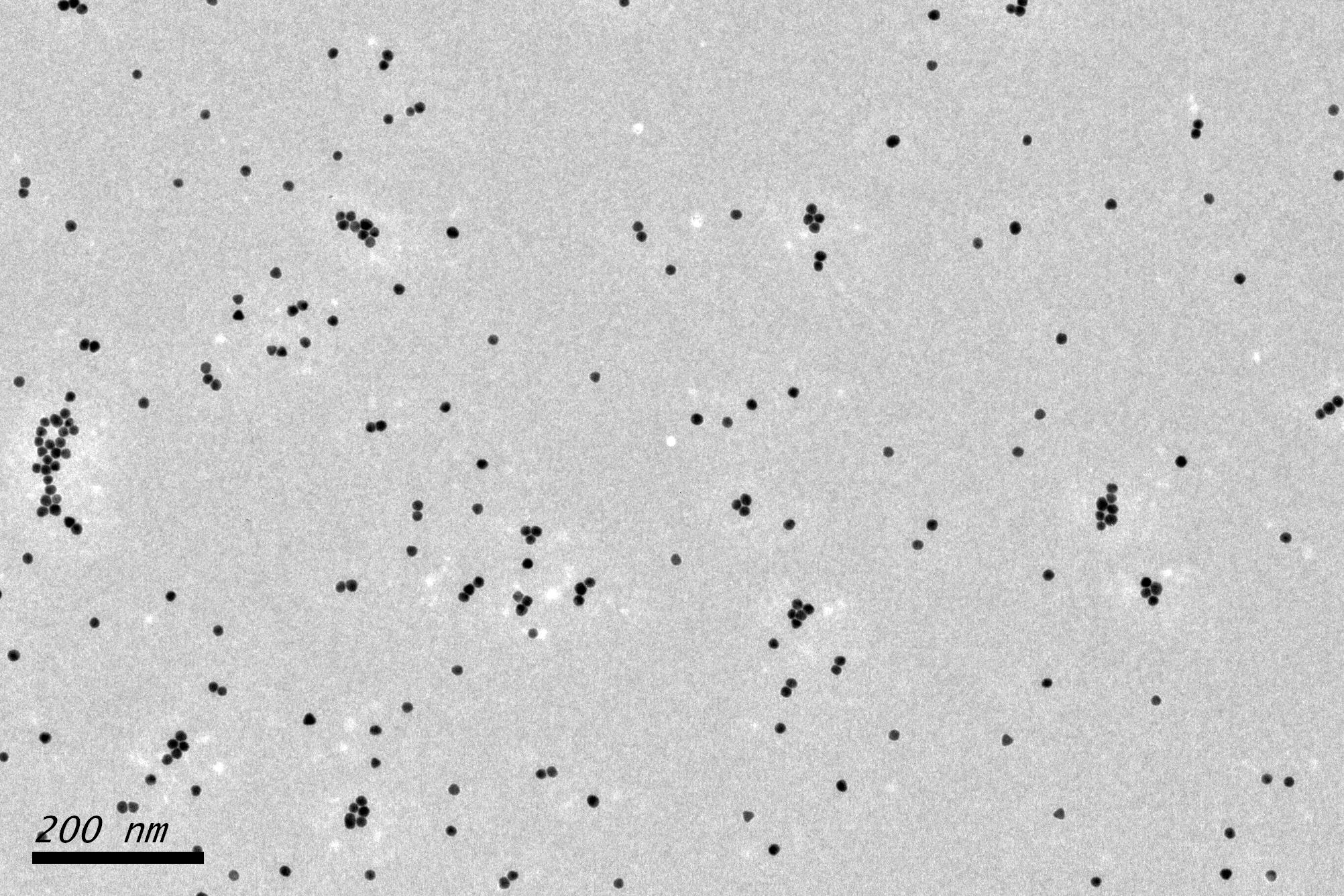

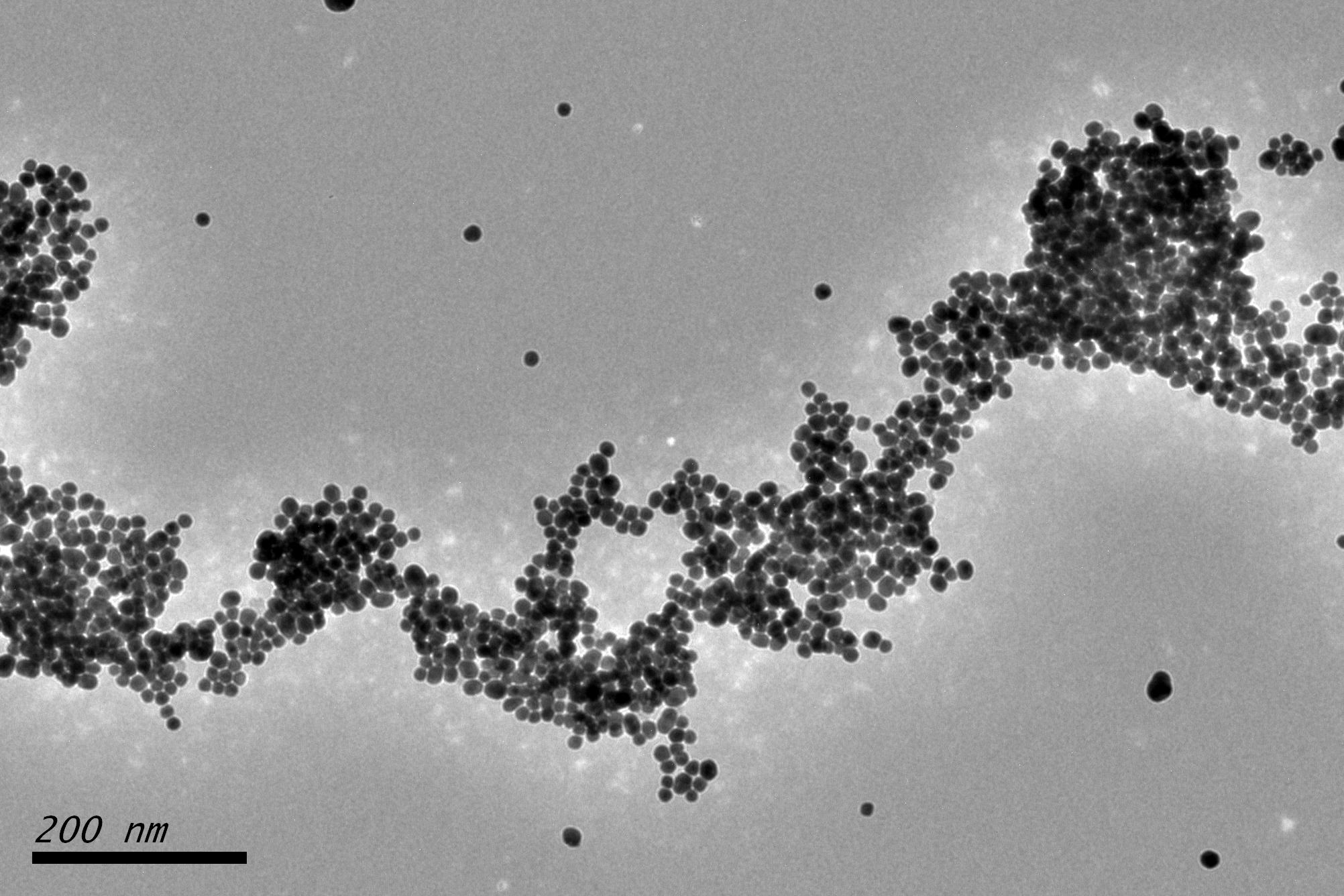


**A**

**B**


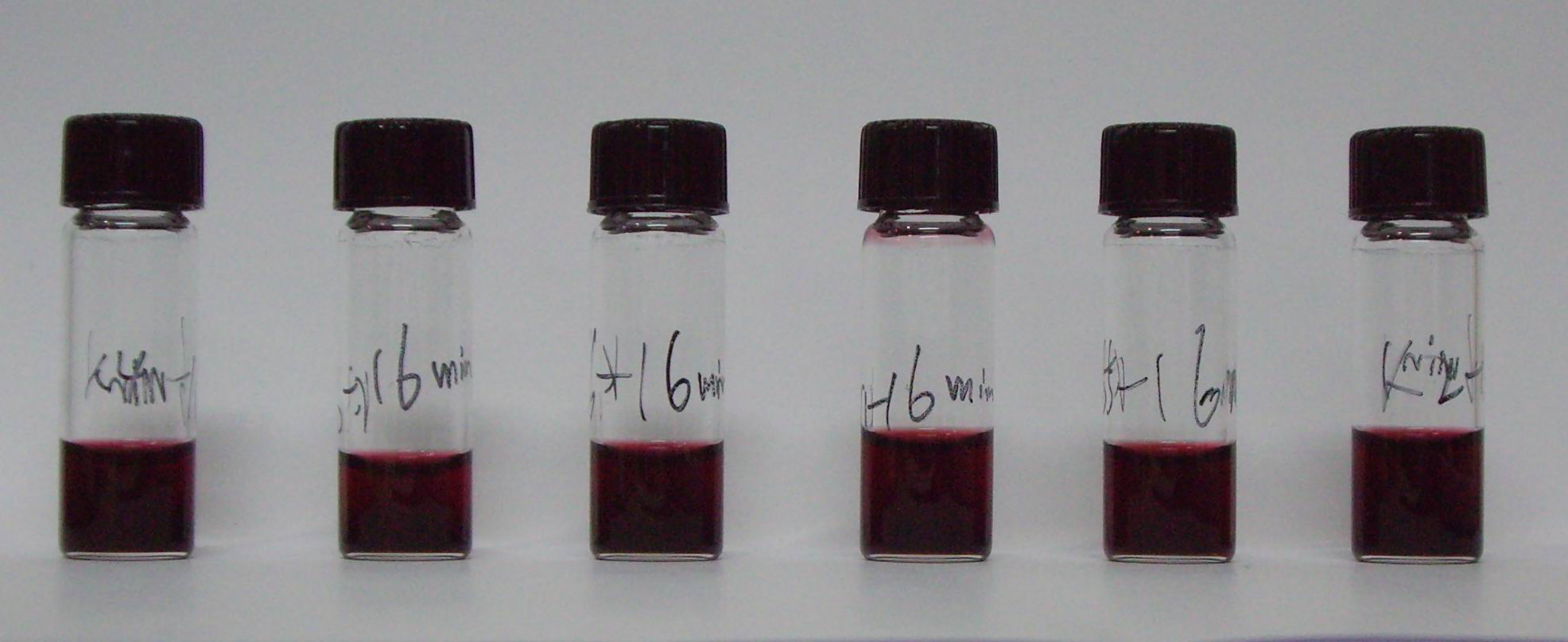


2.2 3.1 3.7 4.4 5.3 6.6

**C**


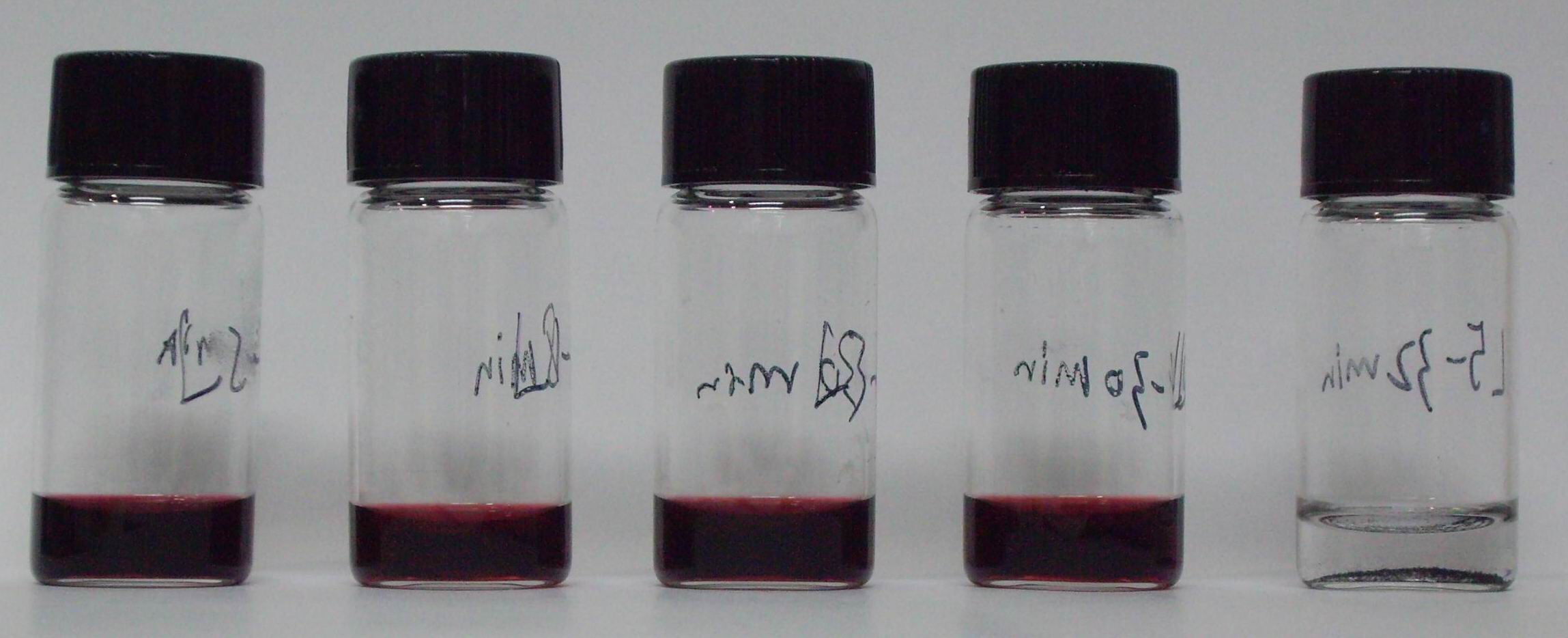


5.5 6.6 7.7 8.8 9.9

**Figure S2.** SPR peak positions of the obtained Au colloids which were prepared in presence of marked NaOH concentration and were taken from the reaction mixture in boil state (A), at 85 ^o^C (B) and at 70 ^o^C (C) at different reaction time and then were naturally cooled.

B

A

C

**Figure S3.** Time dependent UV-vis spectra (left) and corresponding photos (right) of the “frozen” samples which were taken from the reaction flask after different reaction time and were immediately transferred into ice-cooled water. The temperature in all reactions was 85 ^o^C and the concentration of NaOH was 6.0 mM (A), 7.7 mM (B), 9.5 mM (C), respectively.


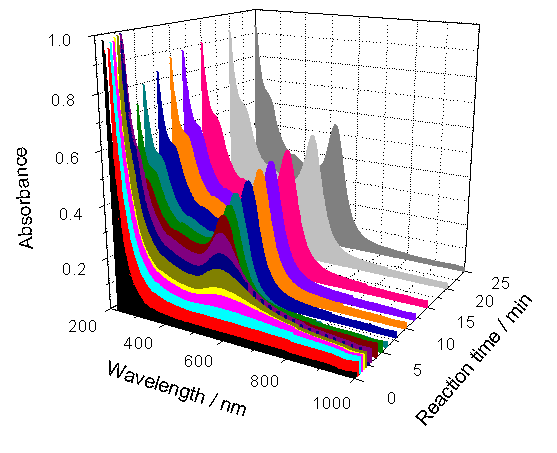

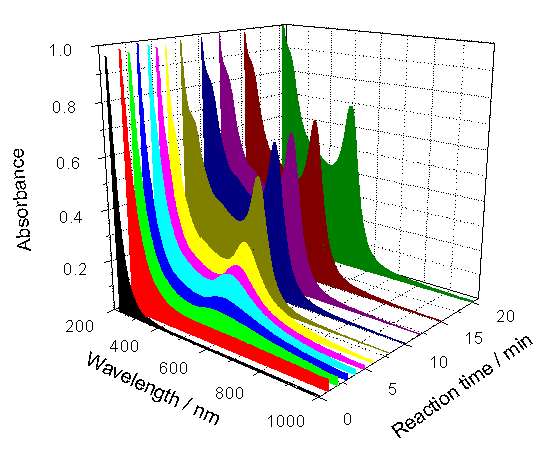

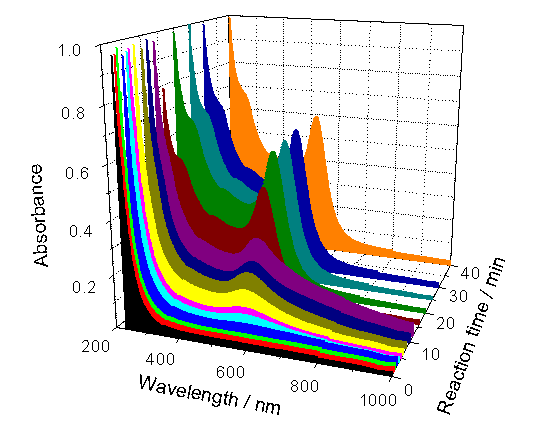


A


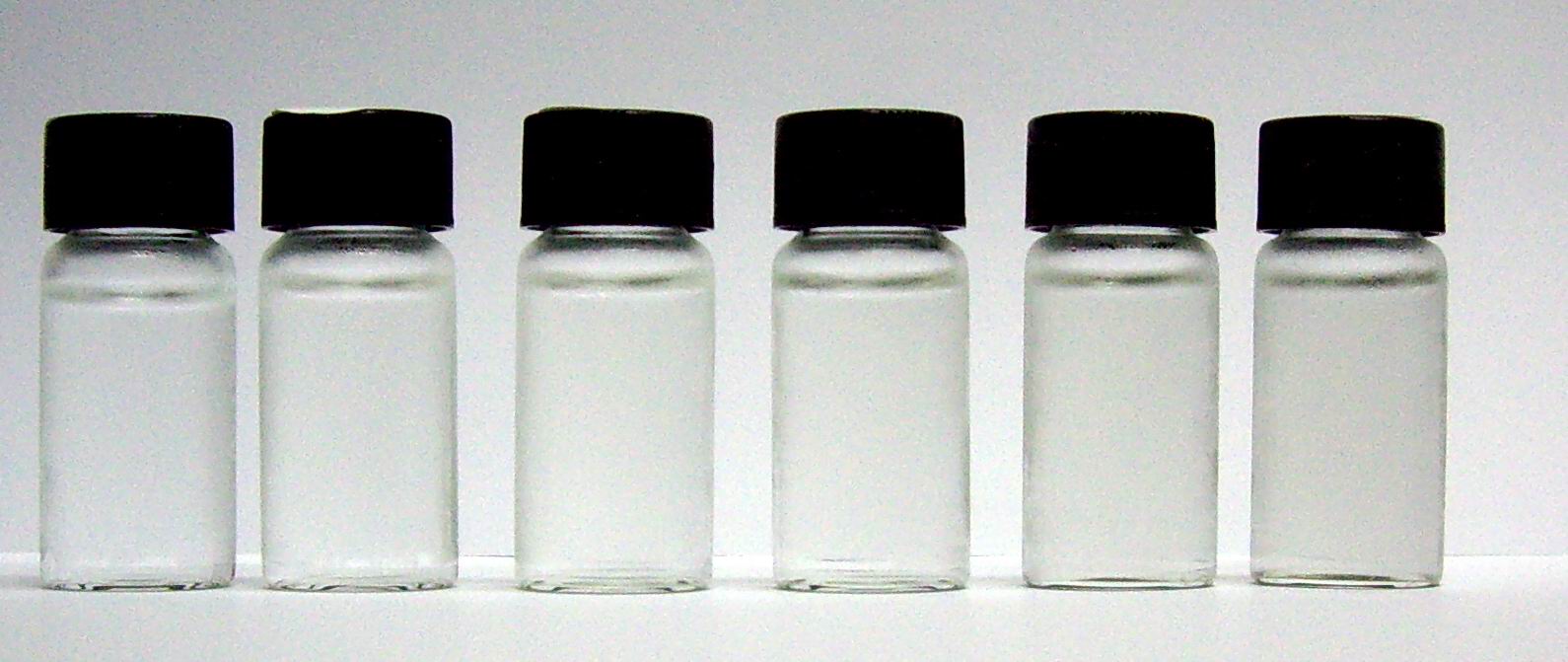

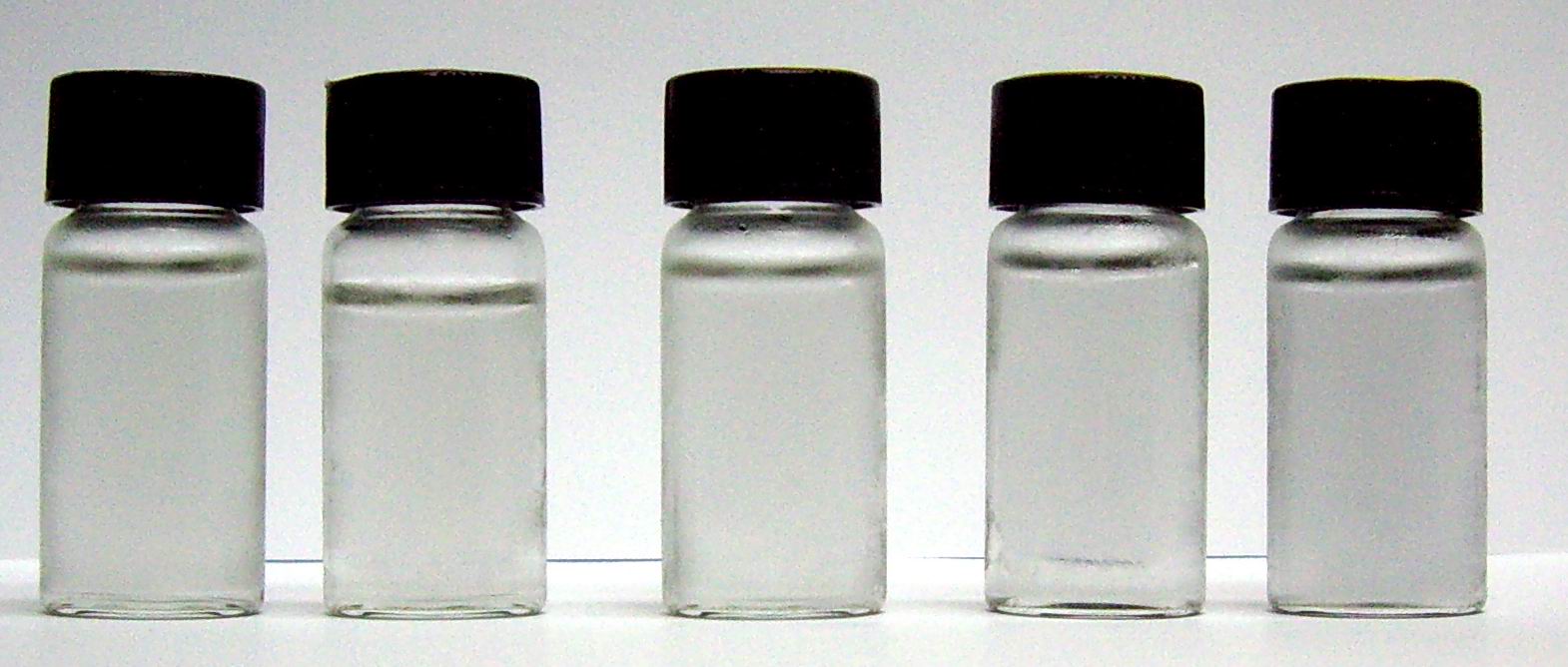


**10s 20s 30s 40s 50s 60s 70s 80s 90s 100s**


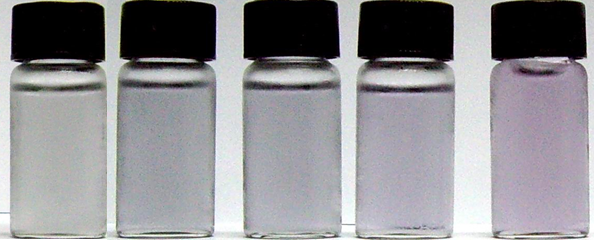

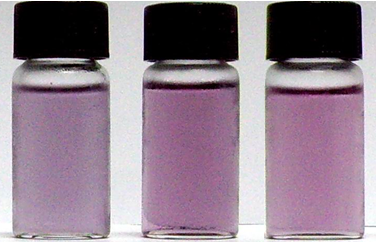

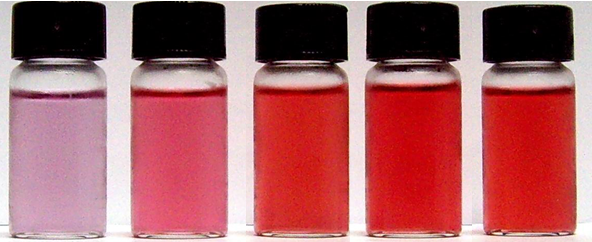

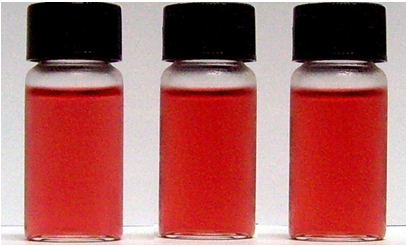


**110s 130s 150s 180s 210s 240s 270s 300s**

**360s 420s 480s 540s 600s 720s 900s 1200s**


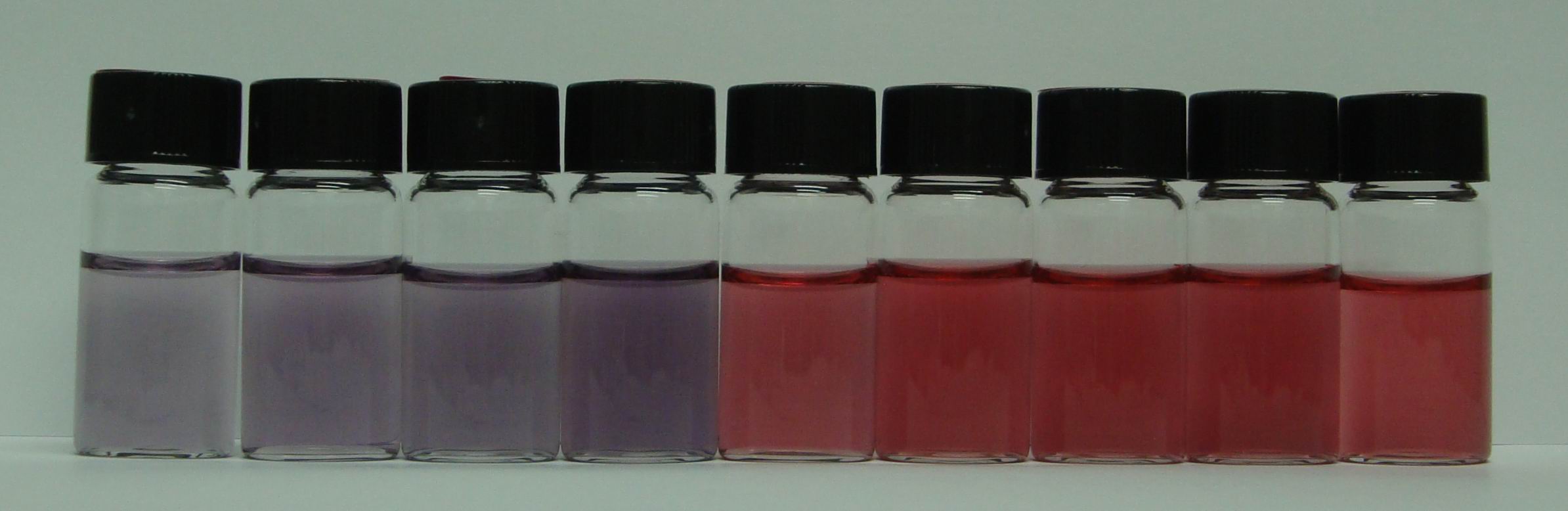

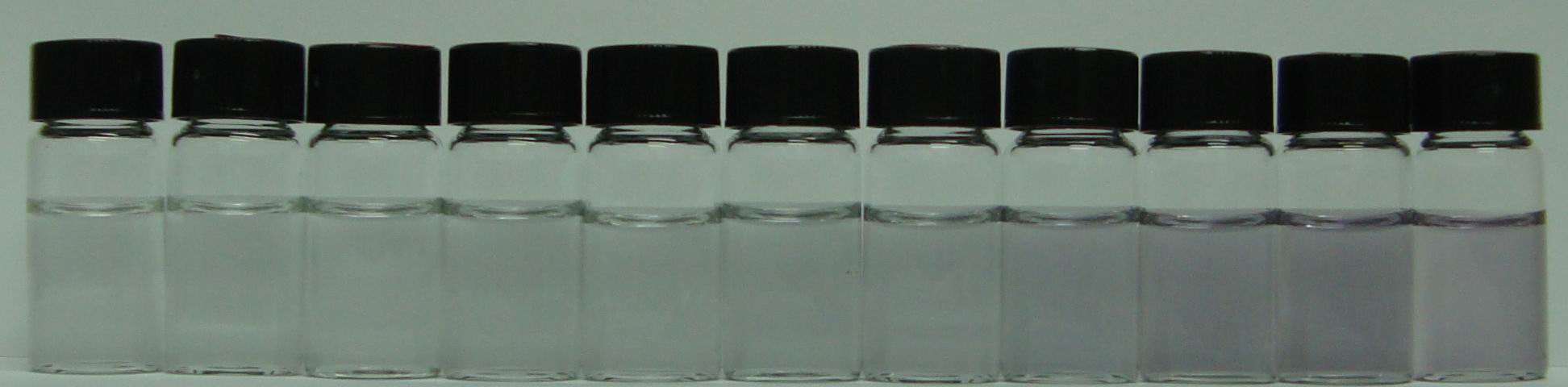


**10s 30s 60s 90s 120s 150s 180s 210s 240s 270s 300s**

**360s 480s 600s 720s 900s 1200s 1500s 1800s 2400s**

B

C


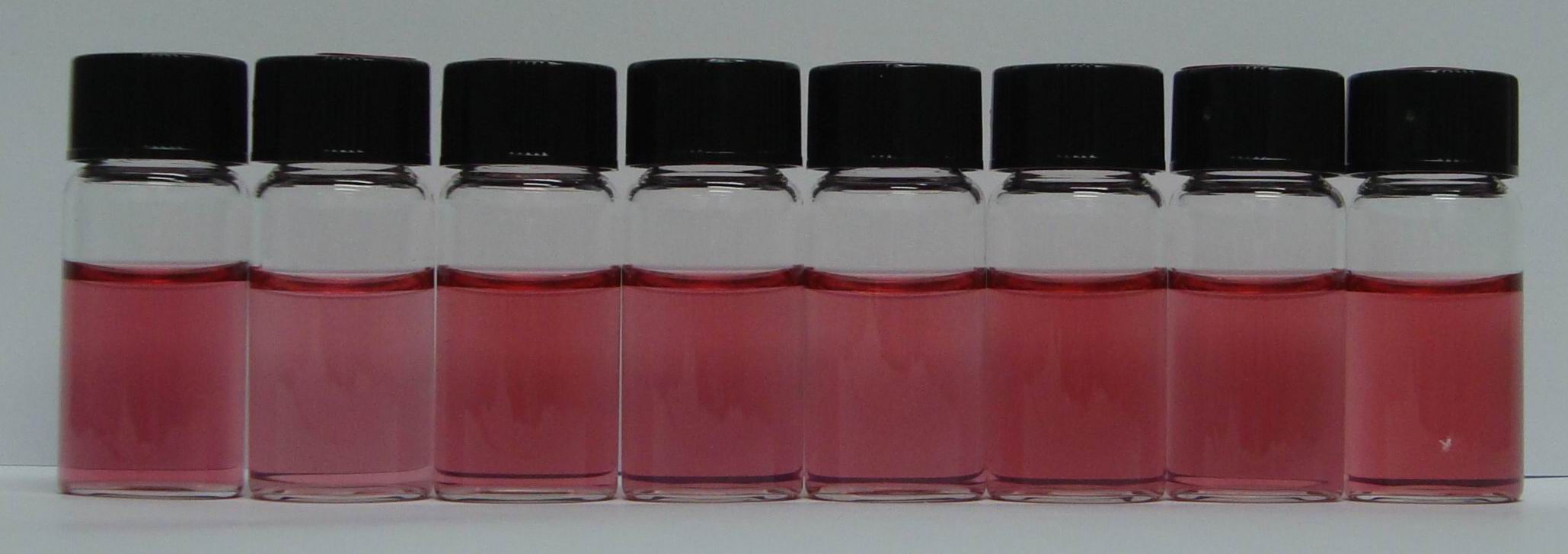

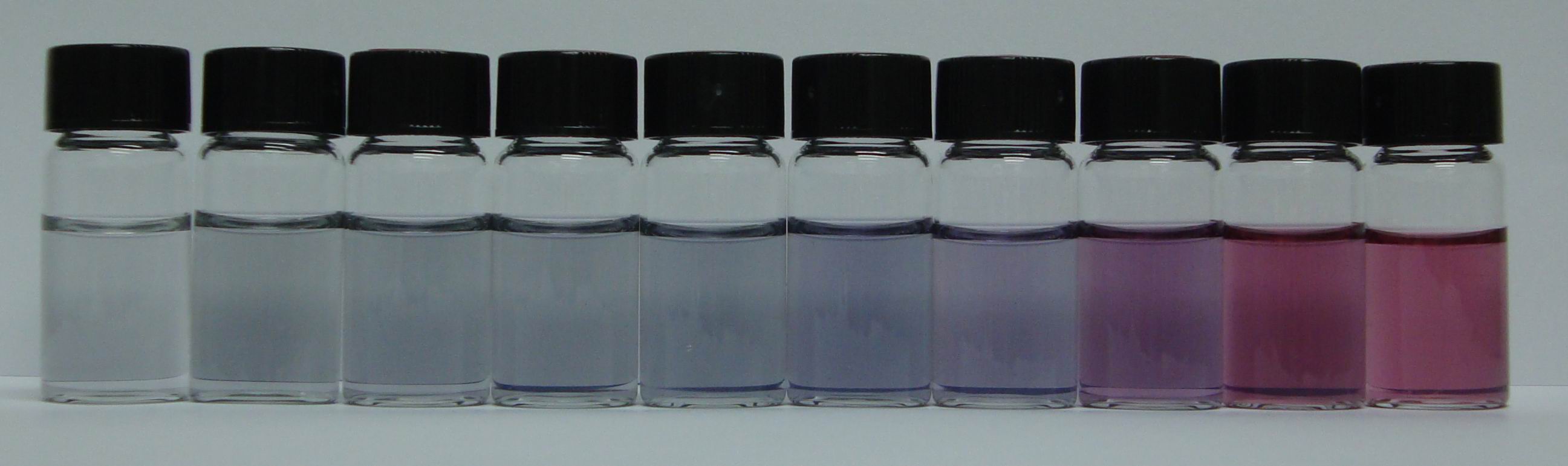


**10s 30s 60s 90s 120s 150s 180s 210s 240s 270s**

**240s 300s 360s 480s 600s 720s 900s 1200s**
